# Fresh red blood cells transfusion protects against aluminum phosphide-induced metabolic acidosis and mortality in rats

**DOI:** 10.1371/journal.pone.0193991

**Published:** 2018-03-28

**Authors:** Nastaran Rahimi, Amir Hossein Abdolghaffari, Alireza Partoazar, Nina Javadian, Tara Dehpour, Ali R. Mani, Ahmad R. Dehpour

**Affiliations:** 1 Experimental Medicine Research Center, Tehran University of Medical Sciences, Tehran, Iran; 2 Department of Pharmacology, School of Medicine, Tehran University of Medical Sciences, Tehran, Iran; 3 Medicinal Plants Research Center, Institute of Medicinal Plants, ACECR, Tehran, Iran; 4 The Whittington Hospital NHS Trust, London, United Kingdom; 5 UCL Division of Medicine, University College London, London, United Kingdom; Massachusetts Institute of Technology, UNITED STATES

## Abstract

**Background:**

Aluminum phosphide (AlP) is used as pesticide in some countries for protection of stored grains. Human poisoning with AlP due to suicide attempt or accidental environmental exposure is associated with very high mortality partially due to development of severe metabolic acidosis. Previous studies have shown that hemoglobin has high buffering capacity and erythrocytes can potentially be used for management of metabolic acidosis. The aim of this study was to evaluate the effect of fresh packed red blood cells (RBC) transfusion on survival and cardiovascular function in AlP-poisoned rats.

**Methodology/Principal findings:**

Rats were poisoned with AlP by gavage. Fresh packed RBC was transfused via tail vein after AlP administration. Acid-base balance, vital signs and mortality was assessed and compared in experimental groups. Infusion of fresh packed RBC (1.5 ml) one hour after AlP (4–15 mg/kg) intoxication was associated with a significant decrease in mortality rate. Packed RBC infusion improved blood pH, HCO_3_^-^, Na^+^ and Ca^2+^ levels. Plasma troponin level was also reduced and ECG changes were reversed following packed RBC infusion in AlP intoxicated rats.

**Conclusions:**

Our results showed that fresh RBC transfusion could ameliorate metabolic acidosis and enhance survival in AlP-poisoned rat. We assume that an increase in pool of RBCs may modulate acid-base balance or potentially chelate AlP-related toxic intermediates via phosphine-hemoglobin interaction.

## Introduction

Aluminum phosphide (AlP), also known as rice tablet, is used as pesticide in some countries for protection of stored grains [1, 2]. Due to accidental exposure or suicide attempts, AlP poisoning is still reported around the globe [3–6]. After exposure with moisture or gastric hydrochloric acid, AlP is decomposed to form phosphine gas (PH_3_) which is a highly toxic compound [7]. Several studies have shown that phosphine exerts severe deleterious effects on biological systems. Singh et al. (2006) reported that phosphine interrupts mitochondrial oxidative phosphorylation via inhibition of cytochrome-c oxidase which leads to anaerobic metabolism, severe metabolic acidosis, multi-organ dysfunction and death [8]. Despite supportive management and intensive care, most of intoxicated patient die following AlP ingestion as there is no specific antidote for AlP poisoning. In 2001, Alter et al. reported a patient who developed metabolic acidosis, progressive heart failure, hypotension and ventricular tachycardia as a result of AlP ingestion [9]. Jaiswal et al. (2009) reported the importance of metabolic acidosis in mortality of AlP by showing that correction of metabolic acidosis can increase patients’ survival by 27% [10].

Due to the importance of metabolic acidosis in pathogenesis of AlP poisoning, the maintenance of plasma pH seems to be a vital step in clinical management of patients with AlP intoxication. Sodium bicarbonate can be administered to increase circulatory pH based on calculation of base deficit [11]. However, severe metabolic acidosis in AlP-intoxicated patients usually requires high doses of bicarbonate that are associated with side effects such as hypercapnia and sodium overload. Red blood cells (RBCs) are involved in extracellular acid-base homeostasis. Hemoglobin has strong buffering capacity and erythrocytes anion-exchange transporters facilitate the transport of ions across RBC membrane [12, 13]. These properties enable RBCs to exhibit great capacity in maintaining blood pH in health (S1 Fig) and disease [14–16]. In addition to modulation of pH, an increase in RBC number has the potential to chelate phosphine gas via phosphine-hemoglobin interaction [17] and thus prevent inhibition of mitochondrial respiratory chain and anaerobic metabolism. We hypothesize that an increase in number of RBCs can prevent development of severe metabolic acidosis during AlP poisoning and the present study reports our investigations on the effect of RBC transfusion on survival, acid-base balance and cardiac function in a rat model of AlP intoxication.

## Materials and methods

### Animals

Wistar rats weighing 200–250 g (8-week-old) were obtained from Department of Pharmacology, Tehran University of Medical Sciences. Animals were housed under standard laboratory conditions in standard polycarbonate cages that included controlled ambient temperature (23±1 °C), a 12-h-dark/12-h-light cycle, and free access to standard animal chow and water.

The study was conducted in accordance with the Guidelines for the Care and Use of Laboratory Animal Ethics Committee of Tehran University of Medical Sciences as well as the National Institutes of Health (NIH publication NO. 85–23; revised 1985). This study was registered and approved by the Tehran University of Medical Sciences Ethics committee (registration number: IR.TUMS.REC.1394.2119). Overall 22 experimental groups were used in this study. Each rat was tested only once and each group consisted of 6–12 animals. All painful procedures including intravenous injection, placement of electrodes for ECG recording were performed under general anesthesia using intraperitoneal injection of ketamine (85 mg/kg, i.p.) and xylazine (15 mg/kg, i.p.). Animals were monitored continuously after administration of AlP and their response to experimental interventions were logged every 30 min. All animals with severe respiratory distress (i.e. pronounced chest movement and gasping through the mouth) were euthanized under carbon dioxide.

### Chemicals

Chemicals were obtained from the following sources: aluminum phosphide (Samiran Pesticide Formulating Co., Tehran, Iran), ketamine HCl (Gedoon Richter Ltd., Budapest, Hungary), and xylazine HCl (Bayer AG, Leverkusen, Germany), sodium bicarbonate (Sterile solution, Shahid Ghazi Pharmaceutical Co., Tabriz, Iran). All other reagents were purchased from Merck (Germany). AlP pills were dissolved in almond oil (final volume 0. 6 ml) [18] and was orally administered by a feeding needle (gavage). The same volume of vehicle (almond oil) was used and orally administered to the control group.

### Packed RBC preparation

For preparation of fresh packed RBC, blood was obtained from heart of intact deeply anesthetized animals through heparinized syringes. Theses anesthetized rats were then sacrificed using carbon dioxide. After performing cross-match, the obtained blood samples were centrifuged at 200 × g at room temperature for 10 min. Platelet rich plasma were separated, then remained red cells were washed three times in isotonic saline (0.9% NaCl) using centrifugation and re-suspending procedure. Following the last wash, packed cells (with hematocrit of 85%) were separated and used as a fresh preparation for infusion via tail vein using an infusion pump (Model 341B, Sage Instruments, Boston, Massachusetts) for 10 min at 37°C.

### Experimental protocol

#### *A*. Assessment of survival following fresh packed RBC transfusion in AlP poisoned rats

Rats were randomized into different groups. Rats in separate groups received different doses of AlP (4, 7, 12, 15, 21 mg/kg, orally using a feeding needle) then the mortality rate of rats was assessed for 24 hours. Dose of 12 mg/kg was chosen for the next steps and the following experiments were carried out to test the effect of packed RBC infusion on survival in poisoned rats. In all studies, at least 8 rats were used in each group.

**Effect of time of packed RBC infusion on AlP-induced mortality:** Packed RBC (1.5 ml) was infused intravenously 30, 60, 90 and 120 min after oral administration of almond oil (as a control group) or AlP (12 mg/kg) to determine the optimum time for RBC administration.

**Effect of different volumes of infused packed RBC on AlP-induced mortality:** Different volume of packed RBC (0.5, 1 or 1.5 ml) was infused intravenously 60 min after oral administration of almond oil (as a control group) or AlP (12 mg/kg) to find the optimized volume of packed RBC infusion.

**Effect of RBC infusion one hour after intoxication on LD**_**50**_ **of AlP:** Animals infused with packed RBC (1.5 ml) 1 hour after receiving different doses of oral AlP (4–21 mg/kg). The mortality was assessed within 24 hours following AlP administration.

#### B. Effect of infused packed RBC on circulatory pH, electrolytes, troponin and ECG

A separate group of animals were used in this section. Thirty minutes after almond oil or AlP gavage (12 mg/kg), animals were anesthetized with intraperitoneal injection of ketamine (85 mg/kg, i.p.) and xylazine (15 mg/kg, i.p.), and placed on a heating pad to maintain body temperature at 37°C. Then, rats were connected to a bio-amplifier and a data acquisition system to record ECG (PowerLab, ADInstruments, Australia). For ECG recording, needle electrodes were inserted under the skin (lead II position). QRS complexes, PR segments, RR intervals, QT intervals and ST segments were identified and calculated by Chart7 software (ADInstruments, Australia). Blood samples were collected from the heart under anesthesia maximum five hours after almond oil or AlP administration. In case animals’ vital sign deteriorated before five hours after AlP administration, blood samples were collected when rats exhibited severe bradycardia and apnea. Blood pH, P_CO2_, electrolytes and troponin I levels were measured using routine methods.

#### C. Effect on bicarbonate administration on survival of AlP intoxicated rats

In a separate group of animals, isotonic solution of sodium bicarbonate was injected in AlP-intoxicated (12 mg/kg) or control rats and the survival was assessed within 24 hours. The amount of bicarbonate was calculated from the “base deficit” of intoxicated animals using the formula 0.4 × body weight (kg) × base deficit (mM; which was estimated from previous experiment) [11]. The calculated volume (~ 9 ml for an average rat) was injected i.p. in three hours with rate of 3 ml/hour. The reason for i.p. injection was that calculate volume exceeded maximum dose volume allowance for intravenous injection in rats.

### Statistical analysis

Data are shown as the Mean ± S.E.M. Overall survival were assessed using Kaplan-Meier test with log rank (Mantel-Cox) was used to determine the significance of mortality rate. One-way analysis of variance (ANOVA) followed by Tukey or Bonferroni post hoc tests were performed to determine the statistical significance of differences between the experimental means. Values of P<0.05 were considered statistically significant. SPSS statistical software package (version 22) or GraphPad Prism (version 7.03) were used for statistical analysis.

## Results

### Effect of different doses of oral AlP on mortality in rats

**Fig 1:**
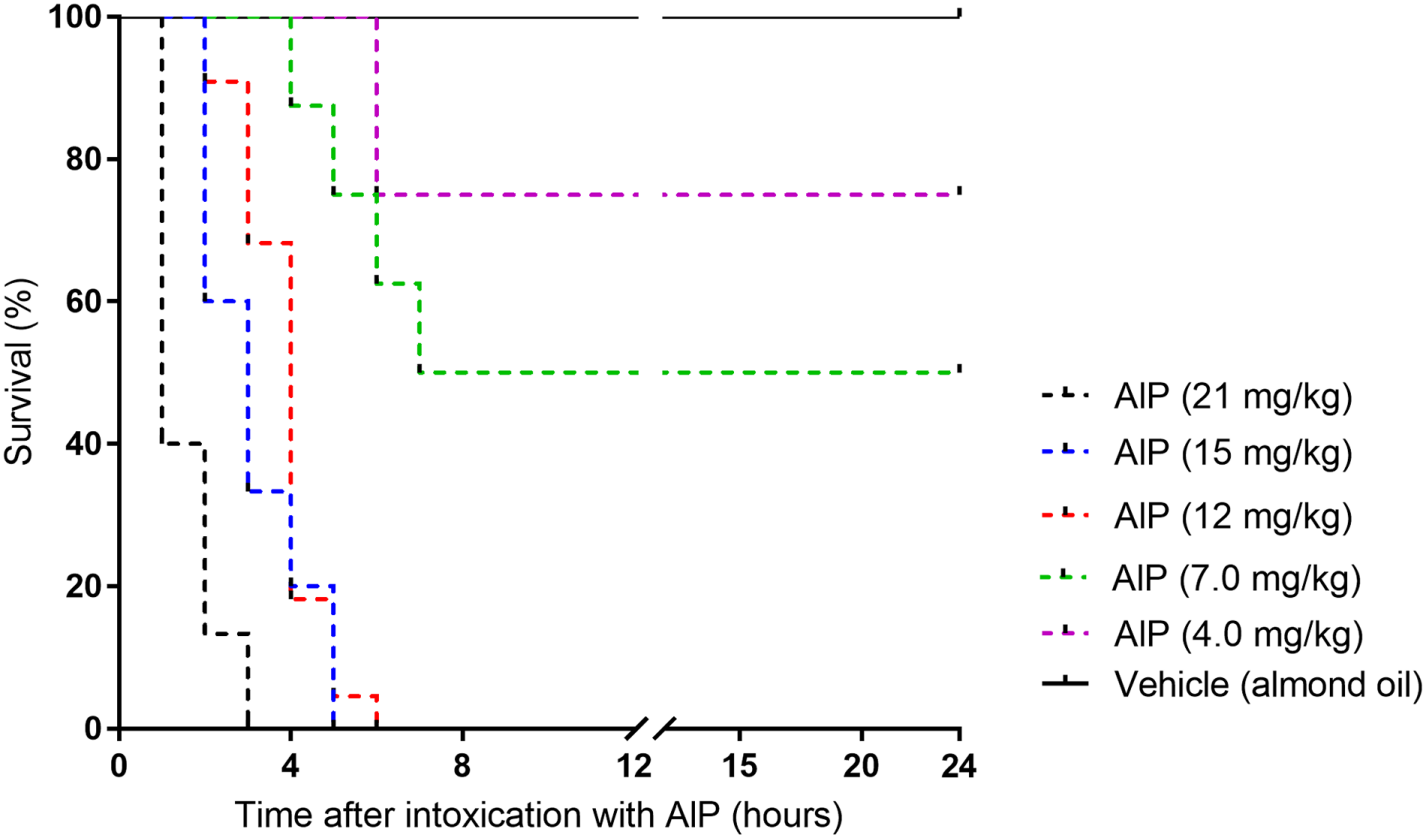
Kaplan-Meier graph showing the survival rate of rats intoxicated with different doses of oral aluminum phosphide (AlP) within 24 hours.

Oral AlP administration showed very high mortality in rats. Doses higher than 7 mg/kg were associated with 100% mortality within 24 hours. Fig 1 shows the Kaplan-Meier graph indicating a dose-dependent increase in mortality. Log-rank test showed that a highly significant mortality at different doses of AlP (P<0.0001). 12 mg/kg was the lowest dose that could induce 100% mortality within 6 hours and this dose was chosen for the next steps of the present study.

### Effect of delay time between AlP intoxication and packed RBC infusion on mortality

**Fig 2:**
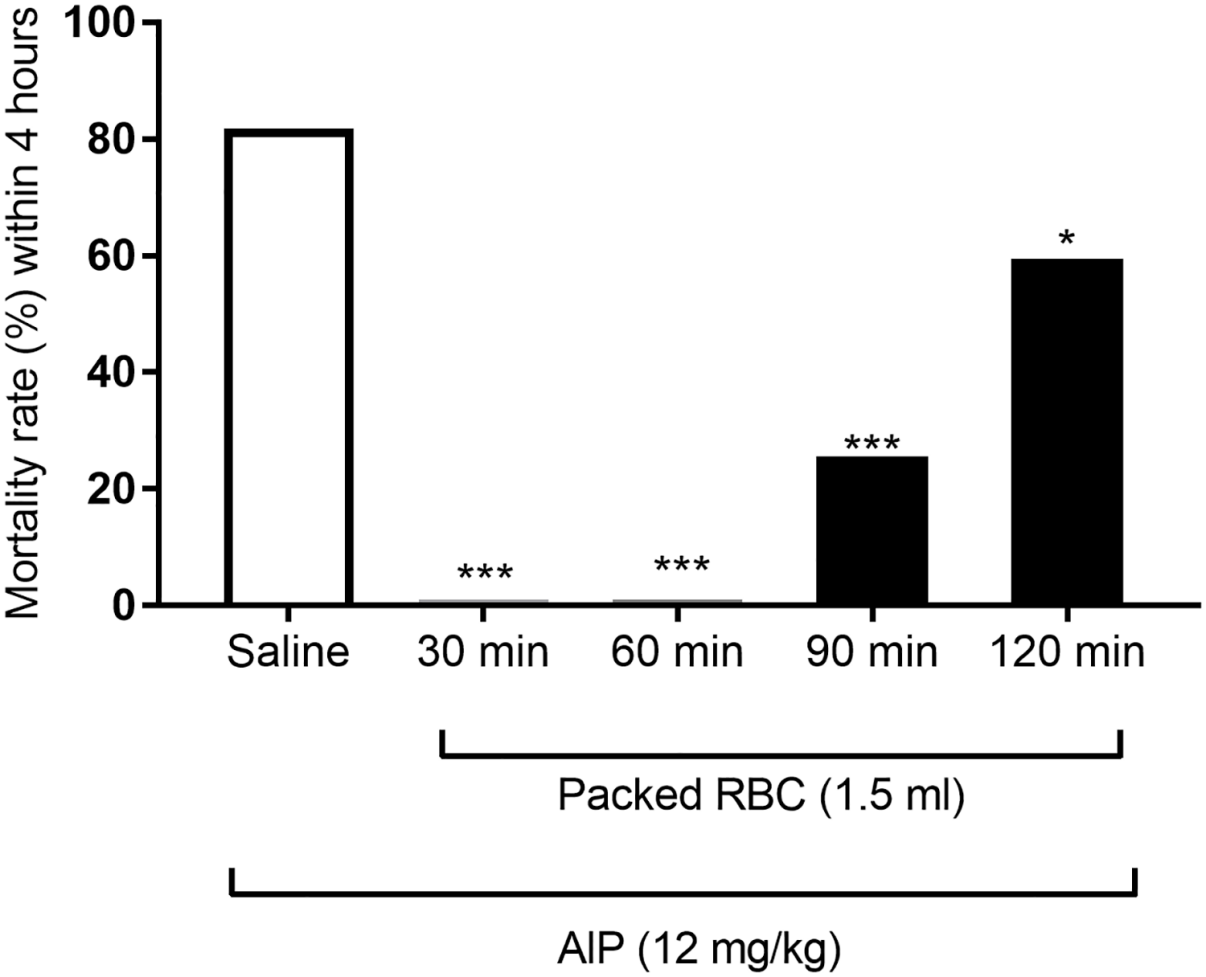
Effects of transfusions of fresh packed red blood cells (RBC) on mortality rate of aluminum phosphide (AlP)-poisoned rats. Packed RBC (1.5 ml) was infused 30, 60, 90 and 120 min after oral administration of AlP (12 mg/kg) and the mortality rate was assessed within 4 hours. * P<0.05, **** P<0.001 compared to saline-treated group.

Fresh packed RBC was infused intravenously 30, 60, 90 or 120 min after administration of almond oil or AlP (12 mg/kg) in rats. As shown in Fig 2, infusion of packed RBC (1.5 ml) at 30, 60 and 90 minutes after AlP administration significantly decreased the mortality rate of poisoned rats (P<0.001, P<0.001, P<0.001 respectively). Mortality rate of poisoned rats decreased by infusion of fresh RBC 120 min after AlP administration but it was significantly more than infusion at 30 and 60 minutes after AlP (P<0.05). None of almond oil treated rats died during experiment.

### Effect of different volumes of infused packed RBC on mortality of AlP-intoxicated rats

**Fig 3:**
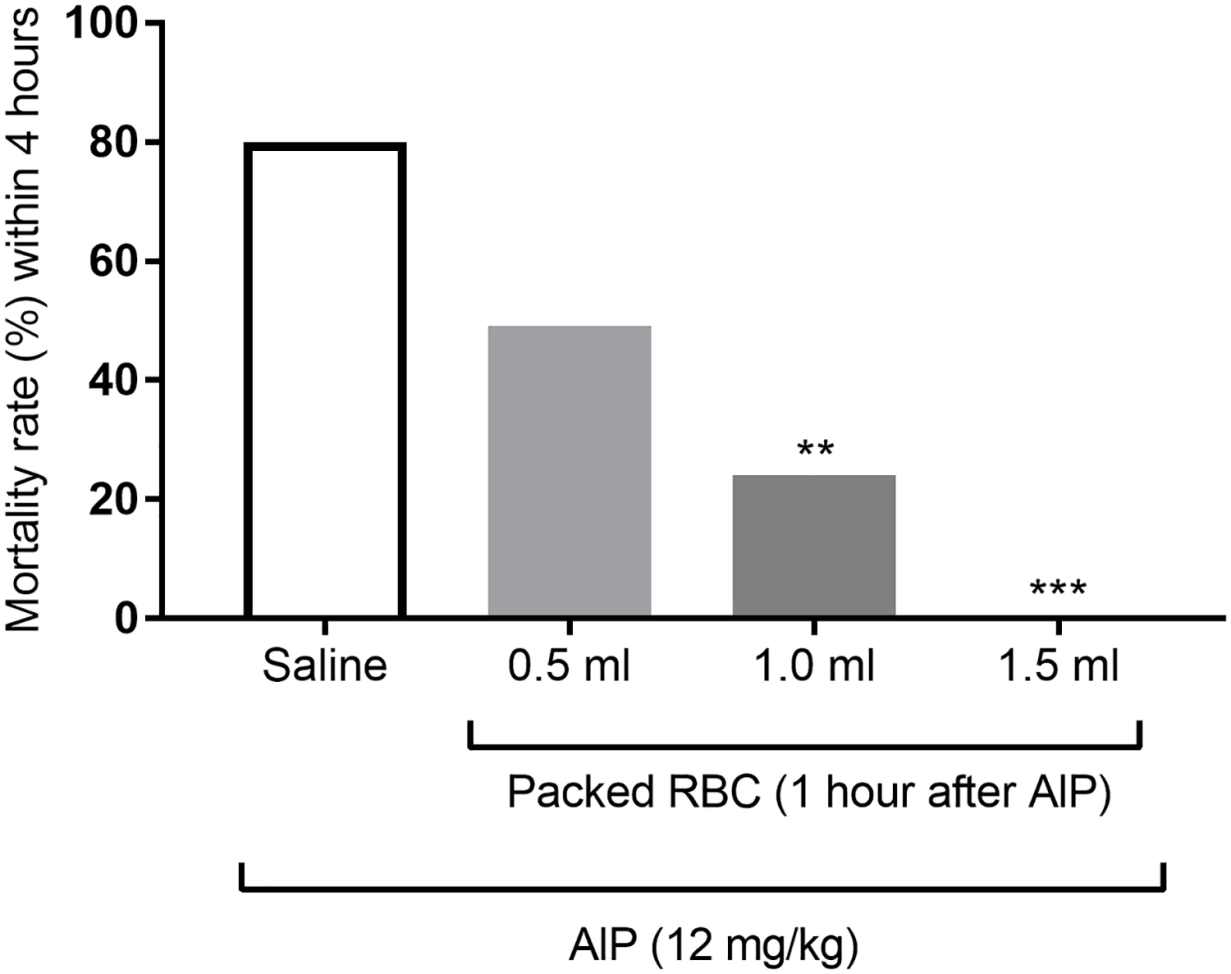
Effects of transfusions of different volumes of fresh packed red blood cells (RBC) on mortality rate of aluminum phosphide (AlP)-poisoned rats. Packed RBC suspensions (0.5, 1 and 1.5 ml) were infused 60 min after oral administration of AlP (12 mg/kg) and the mortality rate was assessed within 4 hours. * P<0.05, ** P<0.01, *** P<0.001 compared to saline-treated group.

Fig 3 illustrates the effect of intravenous administration of different volumes of packed RBC (0.5, 1 and 1.5 ml.) on AlP-induced mortality within 4 hours after drug administration. Administration of packed RBC (1 and 1.5 ml) significantly decreased the mortality rate of animals (P<0.01 and P<0.001) after injection. While, infusion of 0.5 ml packed RBC did not significantly affect the mortality of animals.

### Effect of RBC infusion one hour after intoxication on LD_50_ of AlP

**Fig 4:**
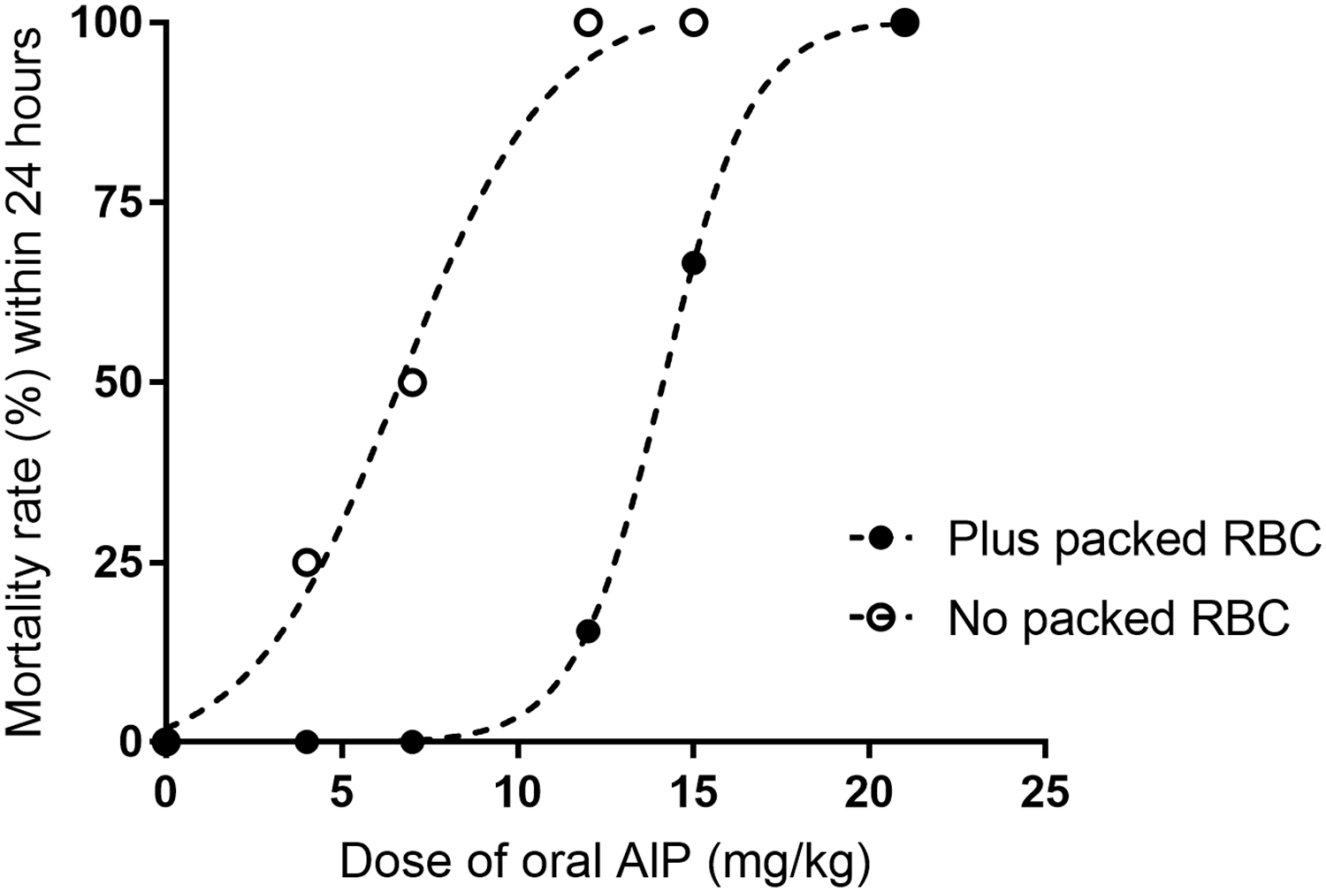
Dose-dependent motility curves for the toxic effect of aluminum phosphide (AlP) in rats with or without transfusion of packed red blood cells (RBC). Packed RBC (1.5 ml) was infused one hour after oral administration of different doses of AlP.

Fig 4 demonstrates dose-response curves for the toxic effect of AlP in two experimental settings. Infusion of 1.5 ml packed RBC, one hour after AlP could double the LD_50_ of orally administered AlP (6.7 mg/kg versus 14.1 mg/kg in AlP and AlP+RBC treated groups respectively). Infusion of packed RBC was unable to decrease mortality when AlP dose was above 15 mg/kg.

### Effect of infused packed RBC on heart rate and ECG parameters

Table 1 shows alterations in heart rate and ECG parameters within four hours in the experimental groups. The following paragraphs summarized this Table:

Heart rate**:** Heart rate significantly decreased over time in AlP-treated rats and treatment with packed RBC (1.5 ml) prevented development of bradycardia over 60–90 minutes in comparison to the AlP and significantly improved within 4 hours.
QT interval**:** QT interval was prolonged over 30–60 minutes in AlP groups in comparison with control group and this change was continued after 60–90 minutes (P<0.001), while this effect was significantly reversed in RBC-treated group.
ST segment**:** Administration of AlP caused a remarkable ST elevation in AlP-treated group in comparison with the control group over 120–150 minutes (P<0.05). ST elevation was significantly continued within 4 hours (P<0.001). Infusion of packed RBC significantly reversed this effect as shown in Table 1.
QRS complexes**:** QRS widening was seen over 120–150 minutes after administration of AlP in comparison to control group (P<0.05). This alteration was significantly continued in AlP-treated rats after 150–180 minutes (P<0.001), whereas treatment with packed RBC infusion significantly reversed these changes.

**Table 1:** Effects of transfusions of fresh packed red blood cells (RBC) on heart rate (HR), and ECG parameters in three experimental groups.

Time (min)	ECG parameter	Control	AlP	AlP + RBC
**0–30**	HR (bpm)	336.33±5.77	318±7.27	318.5±16.95
	QRS (ms)	0.016±0.0010	0.019±0.0036	0.015±0.00084
	QT (ms)	0.067±0.0033	0.068±0.0043	0.070±0.0031
	ST (mV)	-0.031±0.0014	-0.015±0.039	-0.066±0.10
**30–60**	HR (bpm)	332.5±11.19	228.5±60.43	238±11.80
	QRS (ms)	0.015±0.0010	0.018±0.0010	0.017±0.0020
	QT(ms)	0.067±0.0013	0.076±0.0065	0.083±0.0010*
	ST (mV)	-0.033±0.0037	-0.029±0.05	0.023±0.021
**60–90**	HR (bpm)	331.5±11.67	172.83±53.60***	236.5±12.31^#^
	QRS (ms)	0.017±0.00069	0.020±0.0031	0.017±0.0028
	QT(ms)	0.073±0.0021	0.090±0.0072***	0.075±0.00086^###^
	ST (mV)	-0.065±0.0025	-0.039±0.033	-0.024±0.020
**90–120**	HR (bpm)	330.83±10.00	168.5±22.77***	260.33±9.065^###^
	QRS (ms)	0.016±0.0010	0.022±0.0040	0.020±0.0017
	QT (ms)	0.072±0.0023	0.10±0.0074***	0.07±0.0037^###^
	ST (mV)	-0.042±0.015	-0.031±0.039	-0.064±0.032
**120–150**	HR (bpm)	334.16±11.15	142.83±17.34***	275.16±12.01^###^
	QRS (ms)	0.015±0.0010	0.020±0.0024*	0.017±0.0023
	QT (ms)	0.071±0.0034	0.094±0.0015***	0.072±0.0037^###^
	ST (mV)	-0.038±0.012	+0.017±0.013*	-0.096±0.039^###^
**150–180**	HR (bpm)	337.16±4.15	129.5±24.37***	310.33±13.69^###^
	QRS (ms)	0.017±0.00086	0.021±0.0017*	0.016±0.0032^#^
	QT (ms)	0.071±0.0031	0.10±0.0084***	0.073±0.0025^###^
	ST (mV)	-0.05±0.015	+0.035±0.025***	-0.062±0.031^###^
**180–210**	HR (bpm)	332.66±10.84	121.83±29.31***	325±12.81^###^
	QRS (ms)	0.016±0.00098	0.024±0.0026***	0.017±0.0023^###^
	QT (ms)	0.070±0.0033	0.11±0.0041***	0.072±0.0023^###^
	ST (mV)	-0.044±0.015	0.030±0.010***	-0.046±0.023^###^
**210–240**	HR (bpm)	337.66±12.44	98.3±10.31***	333.25±10.55^###^
	QRS (ms)	0.016±0.0041	0.034±0.002***	0.016±0.0043^###^
	QT (ms)	0.070±0.0013	0.12±0.004***	0.070±0.003^###^
	ST (mV)	-0.038±0.02	+0.035±0.0162***	-0.041±0.032^###^

* P < 0.05;

*** P < 0.001 compared to control group;

^#^ P < 0.05;

^###^ P < 0.001 compared to AlP-treated group.

We also assessed development of arrhythmia in experimental rats after administration of either AlP or vehicle (± RBC infusion). All almond oil treated rats had normal sinus rhythm and did not show any evidence for development of ventricular ectopic beats or atrial fibrillation. On the other hand, all AlP treated rats exhibited development of atrial fibrillation starting 84 min (median, range: 76–100 min) after AlP administration. One AlP treated rats showed development of multiple ventricular ectopic beats starting from 85 min post-AlP administration. AlP intoxicated rats that received fresh RBC infusion did not exhibit development of atrial fibrillation or ventricular ectopic beats within 4 hours.

### Effect of infused packed RBC on circulatory blood pH, P_CO2_, plasma electrolytes, and troponin I

Table 2 indicates the effect of AlP (12 mg/kg) with or without packed RBC infusion on blood pH, P_CO2_ and plasma bicarbonate and other electrolytes. A significant drop in blood pH was observed in AlP group which was accompanied with a marked reduction in P_CO2_ and plasma bicarbonate (i.e. consistent with development of metabolic acidosis). All aspects of metabolic acidosis were prevented by infusion of 1.5 ml packed RBC as shown in Table 2. AlP poisoning was also associated with low plasma Na^+^ (P<0.001) and Ca^2+^ (P<0.05) but the level of K^+^ did not change significantly. Infusion of packed RBC (1.5 ml), one hour after AlP intake could significantly reverse the changes observed in plasma Na^+^ and Ca^2+^ (Table 2). In addition to ST elevation (demonstrated in Table 1), AlP administration could raise plasma troponin level significantly (Table 2). Packed cell infusion could prevent both ST elevation and plasma troponin concentrations (Table 2).

**Table 2:** Effects of fresh packed red blood cells (RBC) on blood pH, P_CO2_, plasma electrolytes and troponin I. Values are expressed as mean ± S.E.M.

	pH	P_CO2_ (mmHg)	HCO_3_^−^ (mM)	Na^+^ (mM)	K^+^ (mM)	Ca^2+^ (mM)	Troponin I (ng/ml)
**Control**	7.38 ± 0.02	50.1 ± 1.7	21.01 ± 1.41	148.3 ± 2.7	4.8 ± 0.4	0.98 ± 0.08	0.03 ± 0.002
**AlP**	6.77 ± 0.06***	40.8 ± 1.8**	7.75 ± 1.53***	139.0 ± 1.4***	4.6 ± 0.5	0.88 ± 0.07*	1.03 ± 0.02***
**AlP + RBC**	7.33 ± 0.07^###^	47.7 ± 2.1^#^	20.5 ± 3.08^###^	147.1 ± 4.5^###^	4.9 ± 0.7	1.02 ± 0.07^##^	0.03 ± 0.003^###^

* P < 0.05;

** P < 0.01;

*** P < 0.001 compared to control group;

^#^ P < 0.05;

^##^ P < 0.01;

^###^ for P < 0.001 compared to AlP-treated group.

### Effect on bicarbonate administration on survival of AlP intoxicated rats

**Fig 5:**
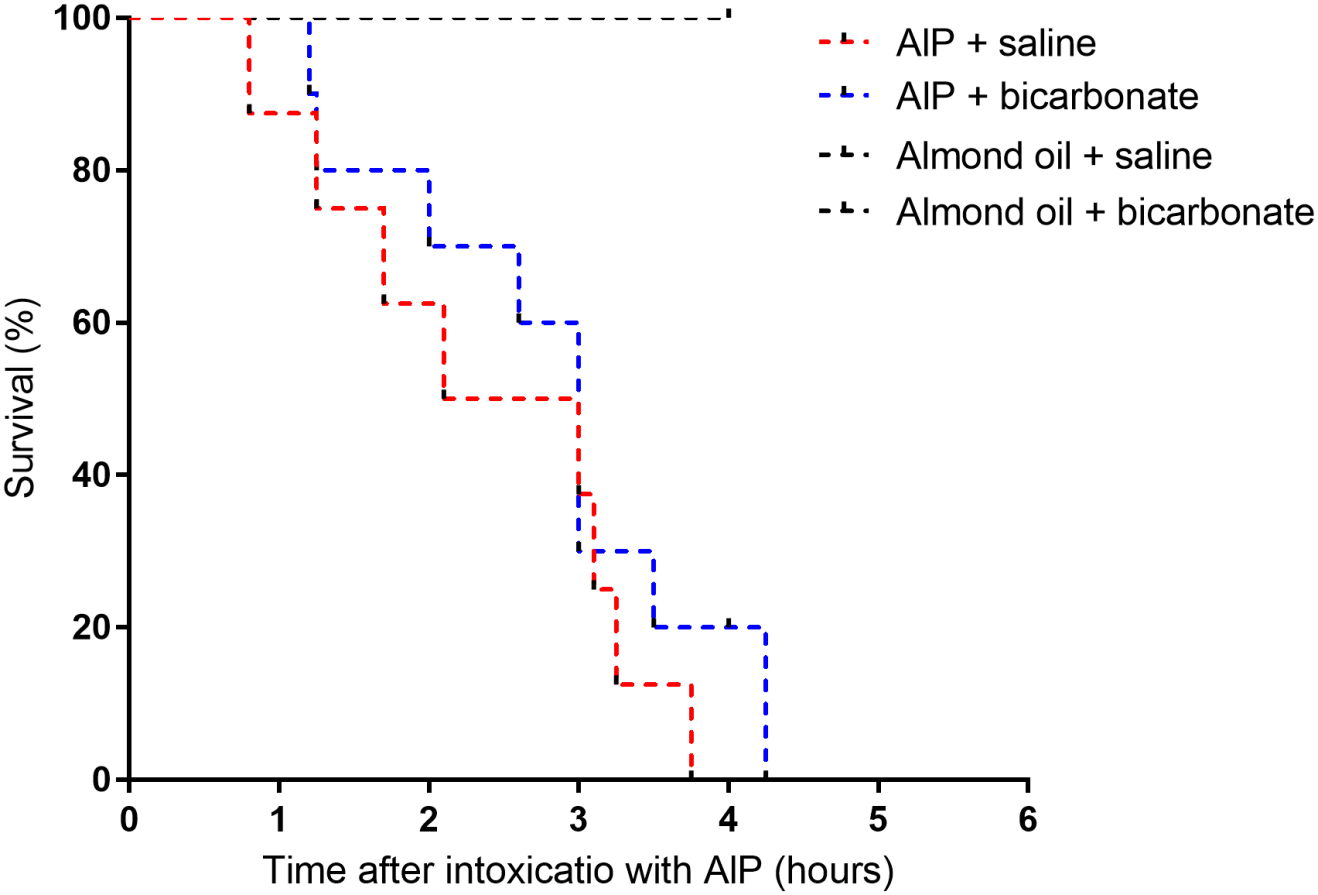
Effect of sodium bicarbonate administration on mortality of aluminum phosphide (AlP) in rats. Kaplan-Meier graph indicates the survival rate of rats intoxicated with AlP with concurrent administration of either isotonic sodium bicarbonate or saline.

Fig 5 shows the Kaplan-Meier graph indicating the effect of either saline or isotonic sodium bicarbonate administration on mortality of AlP intoxicated rats. All AlP treated rats died within 6 hours and sodium bicarbonate therapy could not prevent mortality (P = 0.39, Mantel-Cox test, n = 8–10 in each group). Neither sodium bicarbonate-almond oil nor saline-almond oil injections could induce any mortality as shown in Fig 5. We also collected blood samples when rats exhibited vital signs deterioration (severe bradycardia and apnea). Sodium bicarbonate administration could markedly increase plasma bicarbonate concentration (8.4 ± 0.9 mM versus 28.8 ± 2.6 mM in AlP+Saline and AlP+Sodium-bicarbonate groups respectively, P<0.001). However, sodium bicarbonate administration was unable to significantly increase blood pH (6.94 ± 0.15 versus 7.24 ± 0.08 in AlP+Saline and AlP+Sodium-bicarbonate groups respectively, P = 0.09). P_CO2_ showed a raise following sodium bicarbonate therapy in AlP poisoned rats and mean P_CO2_ was not significantly different from control rats (43.9 ± 3.6 mmHg versus 50.1 ± 1.7 in AlP+Sodium-bicarbonate versus control group, P = 0.34). Analysis of ECG in AlP+Sodium-bicarbonate group showed progressive bradycardia which was accompanied with marked QT prolongation, QRS widening (data not shown).

## Discussion

Aluminum phosphide is commonly used for protection of stored grains in Asian and African countries. Because of its accessibility, it is used during suicide attempts in some countries such as Iran and Morocco [4, 19]. In addition to its use as pesticide, AlP has application in semi-conductor industry and accidental poisoning with AlP has been reported around the globe [3–6]. The mortality of AlP poisoning is very high and clinicians often encounter difficulties in management of severe metabolic acidosis in AlP-poisoned patients. Through the widespread mortality rate of AlP intoxication, still its underlying mechanism is not well understood. It appears that phosphine liberated from AlP in presence of water or gastric acid is responsible for its toxic effects [7, 8, 9]. While, there is no effective known antidote for treatment of AlP; supportive clinical management such as gastrointestinal lavage, intravenous infusion of crystalloids, bicarbonate, calcium gluconate, magnesium sulfate, vasopressors, glucose/insulin, albumin and oxygen therapy are not sufficient to improve survival [7, 20, 21]. There are recent case reports that indicate potential beneficial effects for new experimental therapies in treatment of AlP poisoning but most of these reports are not randomized controlled trials and their efficacy awaits further confirmation [22–28]. We hypothesized that infusion of packed RBC may ameliorate metabolic acidosis, organ failure and mortality during AlP poisoning and our hypothesis was rooted in the following previous reports: *a*. Hemoglobin has a high capacity in buffering the proton (H^+^) and erythrocytes can modulate plasma pH via cooperation between hemoglobin and membrane anion-exchange transporters [12, 13] and (S1 Fig). b. Hemogolbin is a heme protein and can interact with phosphine gas [17,29]. Phosphine gas is liberated by chemical decomposition of AlP in aqueous solution and is highly toxic due to inhibition of mitochondrial heme proteins (e.g. cytochrome-c oxidase) and chelation of phosphine by another heme protein may have potential benefit for the poisoned subjects. In this study, we challenged poisoned rats with freshly prepared packed RBC and the results demonstrated a positive effect in this rat model of AlP-induced intoxication. It appears that interaction of phosphine with hemoglobin in poisoned patients may have implication in prediction of mortality in AlP-intoxicated patients [30–32]. Mashayekhian et al., used non-invasive pulse CO-oximetry in 96 patients with AlP poisoning and demonstrated that carbon monoxide saturation was increased in these patients’ population [30]. Since this finding was not confirmed when tested by standard methods for carboxyhemoglobin measurement, they assumed that hemoglobin was modified by phosphine to form a dyshemoglobin that interfered with pulse CO-oximetry absorption spectra. Nevertheless, this artefactual increase in pulse CO-oximetry signal was an independent predictor of mortality in AlP-intoxicated patients [30]. Another clinical report demonstrated that two patients with G6PD (glucose-6 phosphate dehydrogenase) deficiency and extensive hemolysis, survived AlP poisoning despite having signs and symptoms of fatal toxicity [32]. These reports are in line with a role for phosphine-hemoglobin interaction in pathophysiology of AlP toxicity. Infusion of fresh RBC in AlP intoxicated rats may modulate phosphine-hemoglobin interaction. However, further studies are required to shed light on the mechanism of such modulation.

Our findings demonstrated that administration of fresh packed RBC significantly increased survival rate and prevented development of severe metabolic acidosis in an experimental model. Furthermore, packed RBC transfusion could ameliorate abnormal ECG changes (e.g. ST elevation, QT prolongation and QRS widening) and severity of ventricular arrhythmia. Also, the levels of troponin, plasma electrolytes such as Ca^2+^, Na^+^ were normalized following administration of packed RBC. AlP induces acidosis and this leads to cardiac dysfunction, circulatory failure and hypoxia [7, 19, 33]. Evolving data suggest that metabolic acidosis induces negative inotropic and chronotropic effects and cardiac dysfunction [34]. RBC transfusion is used in variety of clinical settings as a common treatment and intervention [35, 36]. Furthermore, some studies have shown that erythrocytes with high capacity of buffering can modulate extracellular pH and decrease acidosis [12, 14]. Previous studies demonstrated that administration of RBC can normalize pH and improves tissue oxygenation [12, 37, 38]. According to our findings, packed RBC transfusion improved hemodynamic parameters in intoxicated rats. Moreover, the results showed that electrophysiological parameters such as QT interval prolongation, ST elevation, QRS complex widening and development of arrhythmia were normalized after administration of packed RBC in comparison with AlP group. This corroborates with previous studies showing that RBC transfusion could decrease myocardial damage and apoptosis in experimental models of myocardial infarction [15, 39].

Cardiac dysfunction and metabolic acidosis are major clinical symptoms in AlP poisoning [40]. Our results showed that acute AlP intoxication induced low blood pH and low plasma HCO_3_^-^ level which indicates development of metabolic acidosis in this rat model. We showed that RBC transfusion could normalize pH and plasma Na^+^ and HCO_3_^-^ which corroborates with improvement of acid-base balance in AlP poisoned rats. We also tested the effect of sodium bicarbonate in our experimental model. Sodium bicarbonate therapy did not increase survival in our model. Moreover, it was unable to restore acid-base balance in AlP poisoned animals. In fact, the raise in pH was minimal and the pattern of changes in pH, plasma bicarbonate and P_CO2_ indicated a mixed acid-based imbalance following sodium bicarbonate therapy in poisoned rats. Severe metabolic acidosis in AlP-treated rats was associated with a high base deficit which required very high dose of sodium bicarbonate. Delivery of such high does may be associated with complications such as sodium overload. We were unable to restore metabolic acidosis with sodium bicarbonate therapy and this finding is in line with most clinical reports for the existence of resistant metabolic acidosis during AlP poisoning [20]. After a pilot study, we were surprised to observe that a simple intervention (fresh packed RBC infusion) had such significant effect. The present study reports our investigations on the effect of RBC transfusion on survival, metabolic acidosis and cardiac function in a rat model of AlP intoxication. We have not explored the mechanism of this effect in present study. Future studies will pave the way to elucidate the protective mechanism of packed RBC infusion in AlP intoxication.

We investigated the effect of different volumes of packed cells on survival. 0.5 ml packed RBC was not enough to prevent mortality in this model and we observed that 1.5 ml could markedly reduce mortality. An average rat with body mass of 300 g has ~19 ml blood [41] and adding 1.5 ml leads to 7.3% increase in total blood volume. In terms of hematocrit, we can estimate that adding 1.5 ml of packed RBC (with hematocrit of 85%) to 19 ml blood (with hematocrit of 42%) would increase the hematocrit to reach 45%. This means that infusion of 1.5 packed cell in our experimental model has the potential to increase the hematocrit by 3%. In comparison with humans, it is known that one unit of packed cell (300 ml) can increase hematocrit to ~1.9% [42]. Therefore, the volume of packed cell administration in our experimental model is comparable with 1–2 units of packed RBC transfusion in an average human. Another important issue for developing an antidote is knowing the maximum effective time lag between poisoning and antidote administration. We assessed the effect of this time lag on mortality and observed that packed RBC administration can be delayed until 60 min following AlP intoxication. A delay of 90 min from AlP gavage till RBC infusion was still effective in a small fraction of intoxicated animals, but ~30% of rats could not survive when RBC was administered with 90 min delay. Although these results show the limitations of this approach in our experimental setting, it can give a realistic view for the design of future clinical trials in humans. Nevertheless, this time-dependent effect predicts that patients who have been diagnosed early are more likely to benefit from such intervention and fresh packed RBC infusion may not be helpful in patients with delayed diagnosis of AlP intoxication. This may pose itself as a limitation for implementation of this method in clinical settings. Another limitation of the present study is that we followed up the experimental groups for 24 hours and only looked at the markers of cardiac function. In addition to cardiac dysfunction, patients with AlP poisoning may suffer from lung, hepatic and multiorgan failure which can complicate the prognosis and clinical management of AlP intoxication. The effect of fresh packed RBC infusion on AlP-induced multiorgan failure awaits further investigations.

### Conclusion

We reported that administration of fresh packed RBC increases the rate of survival in rats intoxicated with AlP. The mechanism of this protective effect is not known but we hypothesize that a significant improvement in acid-base balance may play a role.

## Supporting information

S1 Fig The effect of erythrocytes on the buffering capacity of bicarbonate buffer *in vitro*. To examine the effect of erythrocytes on the buffering capacity of bicarbonate buffer, fresh erythrocytes were prepared and washed three times using isotonic 0.9% saline as described. 3 ml washed erythrocytes were added to 27 ml of either isotonic 0.9% NaCl solution or isotonic physiological buffered solution containing 25 mM sodium bicarbonate. Isotonic HCl solution was prepared by diluting HCl in NaCl solution to make a 10 mM isotonic HCl saline solution (310 mOsmol/kg). A magnetic stirrer was used to study erythrocyte suspensions at room temperature. Isotonic HCl solution was added to the erythrocyte suspensions with a constant rate of 300 μl per 30 seconds and the changes in pH were measured using a digital pH meter (Mettler Toledo, USA). A separate set of erythrocyte-free physiological buffered solution (30 ml) containing 25 mM bicarbonate was also used as control. Each experiment was repeated at least 3 times. The composition of bicarbonate physiological buffered solution in mM was as follows: NaCl, 112; NaHCO_3_, 25; glucose, 10; KCl, 5; CaCl_2_, 1.8; MgCl_2_, 1; NaH_2_PO_4_, 0.5; KH_2_PO_4_, 0.5. Data are expressed as mean ± S.E.M. As shown in S1Fig, both bicarbonate buffer and erythrocyte suspension (in normal saline) resisted against changes in pH induced by addition of HCl *in vitro*. Addition of erythrocytes to bicarbonate buffer significantly enhanced the buffering capacity of both milieus (Two-way ANOVA, P<0.001). This indicate that fresh erythrocytes can markedly enhance the buffering capacity of bicarbonate-based physiological solutions.
(TIF)
