# Supplementary figures and images for: Fresh red blood cells transfusion protects against aluminum phosphide-induced metabolic acidosis and mortality in rats
Source: PLoS One. 2018 Mar 28;13(3):e0193991. doi: 10.1371/journal.pone.0193991 (PMC5874013; doi:10.1371/journal.pone.0193991)

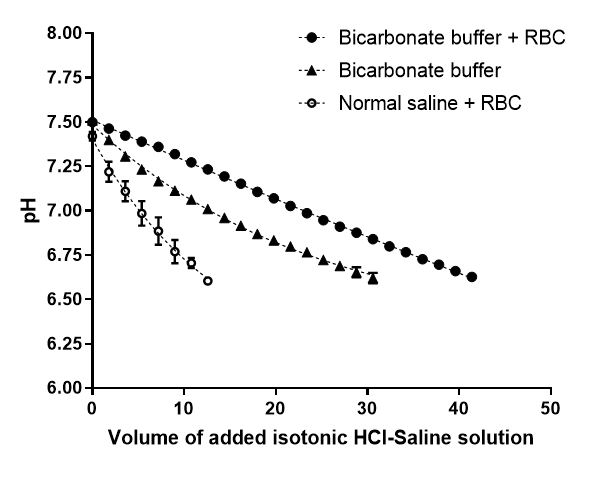

Supplement: S1 Fig — To examine the effect of erythrocytes on the buffering capacity of bicarbonate buffer, fresh erythrocytes were prepared and washed three times using isotonic 0.9% saline as described. 3 ml washed erythrocytes were added to 27 ml of either isotonic 0.9% NaCl solution or isotonic physiological buffered solution containing 25 mM sodium bicarbonate. Isotonic HCl solution was prepared by diluting HCl in NaCl solution to make a 10 mM isotonic HCl saline solution (310 mOsmol/kg). A magnetic stirrer was used to study erythrocyte suspensions at room temperature. Isotonic HCl solution was added to the erythrocyte suspensions with a constant rate of 300 μl per 30 seconds and the changes in pH were measured using a digital pH meter (Mettler Toledo, USA). A separate set of erythrocyte-free physiological buffered solution (30 ml) containing 25 mM bicarbonate was also used as control. Each experiment was repeated at least 3 times. The composition of bicarbonate physiological buffered solution in mM was as follows: NaCl, 112; NaHCO3, 25; glucose, 10; KCl, 5; CaCl2, 1.8; MgCl2, 1; NaH2PO4, 0.5; KH2PO4, 0.5. Data are expressed as mean ± S.E.M. As shown in S1Fig, both bicarbonate buffer and erythrocyte suspension (in normal saline) resisted against changes in pH induced by addition of HCl in vitro. Addition of erythrocytes to bicarbonate buffer significantly enhanced the buffering capacity of both milieus (Two-way ANOVA, P<0.001). This indicate that fresh erythrocytes can markedly enhance the buffering capacity of bicarbonate-based physiological solutions. (TIF) [file pone.0193991.s001.tif]
